# First Insights from On-Board Fish Gutting into the Zoonotic Nematode Burden of Pouting (*Trisopterus luscus*) at the Point of Sale to the Consumer

**DOI:** 10.3390/pathogens14030252

**Published:** 2025-03-04

**Authors:** Francisco Javier Arrebola-Casañas, Mario Garrido, Francisco Javier Adroher, Rocío Benítez, Manuel Morales-Yuste

**Affiliations:** 1 Departamento de Parasitología, Facultad de Farmacia, Universidad de Granada, 18071 Granada, Spainfadroher@ugr.es (F.J.A.); 2 Department of Biology and Geology, Physics and Inorganic Chemistry, Biodiversity and Conservation Area, Rey Juan Carlos University, Móstoles, 28933 Madrid, Spain; mario.garrido@urjc.es

**Keywords:** anisakiasis, *Anisakis*, *Hysterothylacium*, Gadidae, *Trisopterus luscus*, evisceration

## Abstract

A survey was conducted to assess the impact of on-board gutting in the pouting fishery, *Trisopterus luscus* (L.), from the Bay of Biscay (area FAO 27.VIII) on the parasite burden of macroscopic ascaridoid nematodes, including anisakids (causing anisakidosis) and raphidascaridids (causing consumer rejection) in these fish. The fish were caught in the Bay of Biscay and collected from the fish market in Granada (southern Spain). Fish larger than 25 cm were gutted on board after capture. A detailed examination of the fish revealed the presence of nematode larvae, which were identified morphologically and molecularly (PCR-RFLP: polymerase chain reaction with restriction fragment polymorphism). Results revealed that ungutted fish harbored only third-stage larvae of ascaridoids (*Anisakis* and *Hysterothylacium*) while prevalence reached up to 91%. In contrast, gutted fish exhibited a significant reduction in both the prevalence (36%) and mean abundance (MA, 4.44 vs. 0.91) of these larvae. The prevalence of *Anisakis* spp. larvae was reduced by over 20%, with a more pronounced reduction in abundance of more than 40% (MA, 1.56 vs. 0.91). *Hysterothylacium* larvae were completely absent (MA 2.88 vs. 0.00). These findings indicate that gutting, while not highly efficient, lowers *Anisakis* larvae presence, thereby reducing the risk of anisakiasis to consumers. Additionally, the complete removal of *Hysterothylacium* larvae enhances the fish’s appearance, making it more appealing and increasing its commercial value, as well as reducing the risk of seizure by health authorities. Further research on these on-board evisceration practices is needed to enhance effectiveness and reduce zoonotic nematodes in commercial fishes.

## 1. Introduction

Fish is an essential part of the human diet. Therefore, it is necessary to ensure that it reaches the consumer in optimal sanitary and preservation conditions. In recent decades, health authorities have increasingly raised concerns regarding the emergence of anisakiasis in humans. Anisakiasis, or anisakidosis, is an emerging, underdiagnosed, and also neglected disease in many countries, especially those with fewer resources [1]. This condition is acquired through the consumption of insufficiently thermally treated fish harboring viable third stage larvae (L3) of parasitic nematodes belonging to the family Anisakidae, particularly the genus *Anisakis* (>97% of cases) [1,2]. However, at least one case of infection, classified at the time as non-invasive anisakiasis, has been documented with *Hysterothylacium aduncum* [3], a nematode currently classified under the family Raphidascarididae, but previously within the family Anisakidae. Moreover, cases of allergy to these parasites are also considered anisakiasis [4].

At least four species of the genus *Trisopterus* Rafinesque, 1814, can be found in southwestern European markets, with *T. luscus* (Linnaeus, 1758) (called pouting, pout, or bib) being the most common of the Northeast (NE) Atlantic species. These gadids are appreciated by consumers for their delicate meat, yet they deteriorate faster than other gadids, leading to reduced demand. In other regions of Europe, they are also utilized as a source of fish meal [5].

These fish are benthopelagic, typically inhabiting depths ranging between 15 and 400 m. They are predominantly fished in muddy or sandy bottoms at depths between 50 and 200 m on the continental shelf. The diet of this species mainly consists of decapod crustaceans, although it also includes other benthic crustaceans and small fishes [5].

The presence of anisakid nematodes in these fish has been reported on several occasions [6,7,8], but whether the gutting of these fish reduces or eliminates these nematodes and thus the risk of anisakidosis to the consumer has not yet been investigated. However, because the parasite may have migrated into the muscle before the fish is caught, the risk would not be completely eliminated [9,10].

The aim of this communication was to test whether the gutting of pouting (the manual removal of the digestive tract), carried out onboard after capture, led to a reduction in the parameters of infection by anisakids (and raphidascaridids), resulting in a real reduction in the potential risk of anisakidosis from the consumption of *T. luscus*, a fish that can be found in the fish markets of Spain.

## 2. Materials and Methods

### 2.1. Fish Sampling and Parasitological Analysis

**Figure 1:**
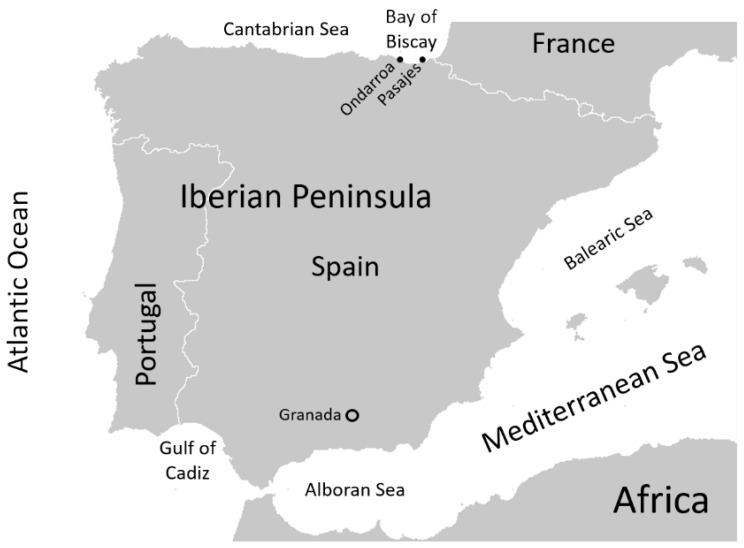
Map of the Iberian Peninsula. The area of investigation showing ports where *Trisopterus luscus* samples were landed (•) and the fish markets sampled (o).

Between December 2021 and November 2022, a total of 45 specimens of pouting, *Trisopterus luscus* (Gadidae), were obtained from the Bay of Biscay as they became available. They occasionally arrive at the fish markets in the city of Granada (southern Spain). Fish are transported on ice from the landing ports of Ondarroa and Pasajes, located on the Basque–Spanish coast of the Bay of Biscay (NE Atlantic, northern Spain) (Figure 1). The fish were acquired and transported on ice sheets to the laboratory, where they were immediately measured (total length to the nearest 0.1 cm) and weighed (to the nearest 0.01 g). The specimens longer than 25 cm were gutted onboard the fishing boats by manual extraction of the digestive tract. After observing the absence of nematodes on the surface due to possible migration from the inside, each fish was dissected to separate the viscera and muscles. The digestive tract was thoroughly examined if present (ungutted fish). The visible macroscopic nematodes were collected and, after washing with saline (NaCl 0.9% *w*/*v*), morphologically identified to genus or species level by light microscopy [11,12]. In order to detect larvae that might have been missed by sight in both the muscles and viscera of the fish, the UV press technique was used [13]. Briefly, the fish flesh is cut into thin fillets that are pressed and frozen until examined. The viscera are only pressed and then frozen. All fish were individually and completely analyzed for both musculature and viscera. Freezing kills the larvae, which, when thawed, fluoresce under UV light and are easily identified [14]. Each nematode was then placed in an Eppendorf tube, labeled, and frozen at −20 °C until processed for molecular identification. The Fulton condition factor (CF), considered an indicator of fish health [15], was calculated using the formula CF = 100 · W/L^3^ [16,17], where W is the weight of the fish and L is the total length of the fish.

### 2.2. Molecular Identification

The molecular identification of isolated *Anisakis* type I larvae (*sensu* Berland, 1961, [11]) was conducted through polymerase chain reaction with restriction fragment polymorphism (PCR-RFLP) analysis. For this, DNA was extracted from each larva with the RealPure kit (Durviz, S.L., Valencia, Spain), following the manufacturer’s instructions. The rDNA fragment of the ITS1-5.8-ITS2 sequence was then amplified using NC5 (forward) and NC2 (reverse) primers [18]. For PCR, a previously described procedure was followed [19]. Amplification was performed according to Morales-Yuste et al. [20]. From the PCR, an amplicon of 900–1000 bp was obtained and subjected to RFLP using *Hinf I* and *Taq I* (Fast Digest, ThermoFisher, Waltham, MA, USA) as restriction enzymes [21]. For correct identification of the larvae, controls of the expected *Anisakis* species were introduced, that is, *A. simplex sensu* stricto (s.s.) and *A. pegreffii*. A 3% agarose electrophoresis was performed, and a characteristic species banding was obtained [22,23]. When the banding pattern for either of the two restriction enzymes (or for both) is the sum of the patterns of both species, for the purpose of this study, such larvae are considered to exhibit a hybrid or recombinant genotype. The same procedure was employed for 16 of the larvae that had been identified morphologically as *Hysterothylacium*, but *Taq I* and *Alu I* (Fast Digest, ThermoFisher, Waltham, MA, USA) were used as restriction enzymes [20]. For better recognition, two controls were used in each gel, one from *H. aduncum* and one from *H. fabri*, corresponding to *Hysterothylacium* species [24,25] previously described morphologically in commercial fish of the genus *Trisopterus* from Iberian waters [7,26].

### 2.3. Infection Parameters and Statistical Study

The results obtained have been used to calculate the parameters prevalence, mean intensity, and mean abundance, as defined by Bush et al. [27]. These parameters were calculated and compared using the free software *Quantitative Parasitology 3.0* developed by Reiczigel et al. [28]. The Fisher exact test was employed to calculate prevalence differences, whereas the bootstrap two-sample *t*-test (with 20,000 repetitions) was utilized to assess mean intensity and mean abundance. Confidence intervals (95%) were estimated using the Clopper–Pearson method. Student’s *t*-test was applied for pairwise comparison of fish length, weight, and condition factor.

## 3. Results

A total of 151 larvae were isolated from the 34 ungutted specimens of *Trisopterus luscus*. Of these larvae, 53 were morphologically identified as *Anisakis* L3 type I (*sensu* Berland 1961 [11]), while the remaining 98 were morphologically identified as L3 of *H. aduncum*. All larvae were isolated from the abdominal cavity, none being found in muscles. A total of 50 *Anisakis* larvae were successfully identified through the use of RFLP: 28 were identified as *A. simplex* s.s., 16 as *A. pegreffii*, and the remaining 6 presented recombinant genetic profiles between the 2 aforementioned species. Lastly, the 16 *Hysterothylacium* larvae randomly collected and subjected to RFLP were confirmed as *H. aduncum*.

Similarly, the examination of the 11 gutted pouting led to the isolation of 10 L3 larvae from the abdominal cavity, all morphologically identified as *Anisakis* type I larvae. No larvae were detected in the musculature. Molecular identification via RFLP revealed four larvae of *A. simplex* s.s., five of *A. pegreffii*, and one recombinant between the two species.

As shown in Table 1, the prevalence of ascaridoid larvae was high in *T. luscus* arrived at the Granada fish market, especially in ungutted fish (91.2%), contrasting significantly (*p* = 0.001) with gutted fish (36.4%). Additionally, a decrease in the parasite intensity to marginally significant values (*p* < 0.07; df = 34; t = 2.041) occurred. When analyzing nematode genera separately, the presence of *Hysterothylacium* larvae in the gutted fish drops dramatically to zero (mean abundance: *p* < 0.005, df = 44, t = 4.904; prevalence: *p* < 0.001). In contrast, although *Anisakis* prevalence and intensity show a noticeable reduction (>20%), the decrease is not statistically significant. Concurrently, the fish gutting led to a reduction of more than 40% in mean abundance, defined as the number of larvae per examined fish, within the sampled population.

Finally, a reduction in the proportion of *A. simplex* s.s. within the total *Anisakis* spp. population in gutted fish has been observed (Table 2). When considering only the two pure species identified, the *A. simplex* s.s. to *A. pegreffii* larvae ratio decreases from 1.75:1 in ungutted fish to 0.8:1 in gutted fish. Similarly, a variation in the mean abundance per species is observed, with a notable decline in *A. simplex* s.s. (0.82 larvae/ungutted fish vs. 0.36 larvae/gutted fish) while maintaining in *A. pegreffii* (0.47 vs. 0.45, respectively).

## 4. Discussion

*Trisopterus luscus* occasionally arrives at the fish markets in Granada (southern Spain), mainly sourced from the ports of the northern coast of Spain, but only specimens larger than 25 cm arrive at the market gutted onboard.

Regarding parasitization, several nematodes have been reported in *T. luscus*, including larval ascaridoids of both the family Anisakidae (as *Anisakis* spp.) and Raphidascarididae (as *Hysterothylacium* spp.), primarily in the abdominal cavity, but *Anisakis* larvae have additionally been detected in the musculature (prevalence 3–5%) [6,7].

Our results show the presence of *Anisakis* L3 type I, which were molecularly identified as *A. simplex* s.s., *A. pegreffii*, and *A. simplex* s.s. × *A. pegreffii* hybrid as well as L3 of *H. aduncum*, in pouting. Although no larvae were found in the musculature in the present study, it is important to emphasize that postmortem migration of larvae from the abdominal cavity to the musculature has been demonstrated in some fishes [9], so the risk is not completely eliminated, especially when considering that only one larva is required to produce symptoms.

When caught, pouting exceeding 25 cm are partially eviscerated onboard by removing the digestive tract. The removal of the viscera extends its commercial shelf life since this fish spoils quickly, and, at the same time, the process eliminates a number of *Anisakis* larvae, thus reducing the risk of anisakiasis for the consumer. Nevertheless, it must be still considered that health risk is not completely eliminated as encapsulated or free larvae may remain on the viscera that have not been removed. Most of the pouting sold in the Granada fish market is sourced from the Bay of Biscay, and those that arrive gutted are all from this area.

Thus, when comparing ungutted and gutted pouting from the Bay of Biscay, a notable reduction in the prevalence of *Anisakis* (22.7% reduction; see Table 1), although it was not statistically significant, likely due to the limited sample size.

The reduction of all infection parameters (Table 1) may be attributed to gutting; however, other factors, such as the size (age) of the gutted fish, could also play a role in the reduction of these parameters [7]. It is important to note that several factors can influence the prevalence and parasite load of a fish population, including changes in feeding habits, death and/or predation of weaker infected individuals, enhancement of the immune response with host maturation allowing parasites to be eliminated, limitation of parasite lifespan in the host, or behavioral changes in infected fish to avoid fishing grounds ([29] and references therein). In any event, Rodríguez-Merayo and Villegas [30] observed no significant variation in the prevalence or intensity of anisakid (probably *Anisakis*) or *Hysterothylacium* larvae in the pouting between 17.6 and 29.5 cm (a range corresponding approximately to our sample). This seems to suggest, even if only partially, that the removal of the digestive tract from larger pouting has led to a real reduction in both the prevalence and the intensity and abundance of *Anisakis* larvae, and consequently to a reduction in the risk of anisakiasis (infection and/or allergy) for consumers.

The results show a notable discrepancy in the proportion of *Anisakis* species between gutted and ungutted pouting. Considering only the two pure species found, a reduction of about 56% in the parasite abundance of *A. simplex* s.s. is observed when the fish is gutted, while the abundance of *A. pegreffii* remains similar (Table 2). We do not know the reason and/or significance of this change. We could speculate that, although randomness cannot be excluded, it could be related to a hypothetical non-random location of the larvae, which occupy different locations depending on the species. However, other possibilities that could be suggested are akin to those already discussed regarding prevalence reduction. It could be speculated that because *A. simplex* s.s. has a greater proteolytic ability [31,32], it could elicit a greater response from the fish, or that the parasite is more sensitive to the host response than *A. pegreffii*. On the other hand, the different size and therefore age of the ungutted or gutted pouting could influence the distribution of *Anisakis* species in the abdominal cavity, although we are not aware of any studies on this.

Since the pathogenicity of *A. simplex* s.s. in humans is greater than that of *A. pegreffii* [33], the greater reduction in the former could hypothetically lead to a further reduction in the potential risk of human infection from ingestion of gutted pouting. In this sense, and considering that, at least in anchovies from the Bay of Biscay, *A. simplex* s.s. has been found to be the most abundant *Anisakis* species in fish muscle [9], the elimination or reduction of this species in gutted fish reduces the potential load of this parasite in the fish flesh if migration occurs, although to our knowledge it has not yet been reported in this fish species.

With respect to *Hysterothylacium*, the prevalence is reduced to zero (Table 1), representing a 100% reduction. This is likely because *H. aduncum* is typically found in the intestinal mesentery; therefore, when the digestive tract is removed, the mesentery is also likely to be removed. This is a commercial advantage; although the zoonotic potential of the genus *Hysterothylacium* is unlikely (since there is only one known case of digestive disturbance associated with the excretion of an adult *H. aduncum* [3]), there is no doubt that these macroscopic larvae cause rejection by the consumer, so their elimination increases the commercial value of the fish as well as the acceptance by the consumer. In addition, a reduction in the number of visible parasites reduces the likelihood of a seizure by the health authorities [34,35]. On the other hand, allergy to these nematodes cannot be completely excluded, since they share allergens with *Anisakis* [36].

Further studies with larger samples are needed to assess whether onboard gutting really reduces the risk of anisakiasis for the consumer. In this sense, Roepstorff et al. [10], working with North Sea herring, *Clupea harengus*, suggested that onboard evisceration was not sufficient to avoid the risk of anisakiasis due to the presence of larvae in the flesh at the time of capture, as demonstrated by other authors [37]. Nevertheless, Ahuir-Baraja et al. [38] reported that evisceration of blue whiting (*Micromesistius poutassou*) at the point of sale to the consumer is also not an effective method for the complete elimination of ascaridoid larvae, at least in this fish, since some larvae have had time to migrate into the muscle of the fish since capture, and although the larval load is significantly reduced, the risk of infection/allergy to these nematodes is not eliminated. Also, Kumas et al. [39] found that the presence of *Anisakis* larvae in the musculature did not vary after industrial gutting of the fish, but after processing that included the removal of the lower part of the hypoaxial musculature (this was shown earlier by Levsen et al. [40]). Thus, in its latest report, EFSA highlights in its conclusions that “Advanced processing techniques for intelligent gutting and trimming operations could have a significant impact on removing parasites from fish in the future“ [41]. However, it should also be noted that gutting procedures on board fishing vessels may lead to the practice of discharging gutting waste directly into the sea, which not only maintains the life cycle of the parasites but also promotes their spread. Although EU regulations attempt to minimize the problem by prohibiting this practice [34], control procedures have not yet been implemented. It has been estimated that, for example, a trawler in Gran Sol can release about 50,000 larvae into the sea through fish waste every working day [42]. The impact on affected areas can be enormous.

## 5. Conclusions

Although the above methods significantly reduce the number of parasites and therefore the risk of infection, the risk can never be completely eliminated. For all these reasons, it is always necessary to insist to the consumer on the appropriate thermal treatment of fish throughout its mass (−20 °C for >24 h or >60 °C for >1 min) through the sanitary campaigns carried out by the health authorities [1,41,43].

## Figures and Tables

**Table 1:** Descriptive parameters of host fish and parameters of *Anisakis* and *Hysterothylacium* infection in *Trisopterus luscus* landed in ports of the Basque–Spanish coast (northern Spain) and sampled in fish markets of Granada (southern Spain).

	Parameters	Ungutted Fish	Gutted Fish
**Host**			
	No. of fish	34	11
*Trisopterus luscus*	Mean total length ± SD	21.6 ± 1.6	27.5 ± 1.5 ****
	(range)	19.0–25.0	25.0–29.5
	Mean weight ± SD	118.12 ± 25.71	247.59 ± 45.54 ****
	(range)	80.32–167.65	178.15–299.10
	Condition factor ± SD	1.154 ± 0.083	1.184 ± 0.084 ^ns^
	(range)	0.960–1.324	1.007–1.268
**Parasites**			
Ascaridoids	Prevalence (%)	91.2	36.4 ***
	CI 95%	76.3–98.2	10.9–69.2
	Mean intensity	4.87	2.50 ^#^
	(range)	(1–24)	(1–4)
	CI 95%	3.42–7.29	1.25–3.25
	Mean abundance	4.44	0.91 *
	CI 95%	3.03–6.71	0.18–1.82
*Anisakis* L3 type I	Prevalence (%)	47.1	36.4 ^ns^
	CI 95%	29.8–64.9	10.9–69.2
	Mean intensity	3.31	2.50 ^ns^
	(range)	(1–12)	(1–4)
	CI 95%	2.00–5.44	1.25–3.25
	Mean abundance	1.56	0.91 ^ns^
	CI 95%	0.82–2.82	0.18–1.82
*Hysterothylacium* *aduncum*	Prevalence (%)	82.4	0.0 ***
	CI 95%	65.5–93.2	0–28.5
	Mean intensity	3.50	0.00
	(range)	(1–18)	-
	CI 95%	2.61–5.36	n.a.
	Mean abundance	2.88	0.00 **
	CI 95%	2.03–4.47	uncertain

Weight in g; length in cm. Prevalence = 100·N/F, mean intensity = A/N, and mean abundance = A/F; where F is the total number of fish, N is the number of infected fish, and A is the number of larvae. SD: standard deviation. CI: confidence interval. n.a.: not applicable. A comparison of length and weight between gutted and ungutted fish by Student’s *t*-test showed high significance (**** *p* << 0.0001) but was not significant when comparing the condition factor. Fisher’s exact test was used to compare infection prevalence between ungutted and gutted fish. Similarly, a two-sample bootstrap *t*-test (with 20,000 replicates) was used to compare mean intensity and mean abundance between ungutted and gutted fish. *** *p* ≤ 0.001; ** *p* < 0.005; * *p* < 0.01; ns, not significant. # *p* < 0.07, marginally significant.

**Table 2:** Ratio between *Anisakis simplex* s.s. and *A. pegreffii* in ungutted vs. gutted *Trisopterus luscus* *.

	Number of Larvae	Ratio of Number of Larvae	Prevalence Ratio (%)	Mean Abundance (Larvae/Fish)	Mean Intensity (Larvae/Infected Fish)
Ungutted fish	28:16	1.75:1	1.2:1 (35.3:29.4)	0.82:0.47	1.75:1
Gutted fish	4:5	0.8:1	1:1 (27.3:27.3)	0.36:0.45	1:1.25

* Results presented as ratio *A. simplex* s.s.: *A. pegreffii* larvae. The hybrid genotype larvae were excluded.

## Data Availability

The datasets generated during and/or analyzed during the current study are all included in this manuscript.
